# Evidence of successful malaria case management policy implementation in Cambodia: results from national ACTwatch outlet surveys

**DOI:** 10.1186/s12936-016-1200-2

**Published:** 2016-04-08

**Authors:** ACTwatch Group, Joe Novotny, Amandeep Singh, Lek Dysoley, Siv Sovannaroth, Huy Rekol

**Affiliations:** Population Services International, 1120 19th St NW Suit 600, Washington, DC 20036 USA; PSI Cambodia (PSK), No. 29, Street 334, Boeung Keng Kang I Chamcar Mon, Phnom Penh, Cambodia; Clinton Health Access Initiative, #7B, Str. 81 and corner Str. 109, Sangkat Boeung Raing, Khan Don Penh, Phnom Penh, Cambodia; Cambodia National Centre of Entomology, Parasitological and Malaria control, House 372, St Monivong Vong, Boeung Keng Kang I, Chamcar Mon, Phnom Penh, Cambodia; School of Public Health, National Institute of Public Health, Phnom Penh, Cambodia

**Keywords:** Anti-malarial, Treatment, Elimination, Outlet, Availability, Market share, Supply, Public, Private sector

## Abstract

**Background:**

For over a decade, Cambodia has implemented a number of policies and innovative strategies to increase access to quality malaria case management services and address the drivers of multi-drug resistance. This paper utilizes outlet survey trend data collected by the ACTwatch project to demonstrate how changes in Cambodian policy and strategies have led to shifts in anti-malarial markets.

**Methods:**

Anti-malarial ACTwatch outlet surveys were conducted in Cambodia in 2009 (June–July), 2011 (June–August) and 2013 (September–October). A census of all outlets with the potential to sell or distribute anti-malarials was conducted within a nationally representative sample of communes. Drug information, sales/distribution in the previous week, and retail price were collected for each anti-malarial in stock. Information on availability of malaria blood testing was also collected.

**Results:**

A total of 7833 outlets were enumerated in 2009, 18,584 in 2011, and 16,153 in 2013. The percentage of public health facilities with at least one anti-malarial in stock on the day of the survey increased between 2009 (65.8 %) and 2011 (90.0 %) and remained high in 2013 (82.0 %). Similar trends were found for village malaria workers (VMW). Overall, private sector availability of anti-malarials declined over time and varied by outlet type. By 2013 most anti-malarial stocking public health facilities (81.5 %), VMW (95.4 %), private for-profit health facilities (64.8 %), and pharmacies (71.9 %) had the countries first-line artemisinin-based combination therapy (ACT) treatment in stock. In 2013, 60 % of anti-malarials were delivered through the private sector, 40 % through the public sector, and the most common anti-malarial to be sold or distributed was the first-line ACT, comprising 62.8 % of the national market share. Oral artemisinin monotherapy, which had accounted for 6 % of total anti-malarial market share in 2009, was no longer reportedly sold/distributed in 2013. Malaria blood testing availability remained high over time among public facilities and VMW, with availability over 90 % in 2011 and 2013. Moderate availability was observed in the private sector.

**Conclusions:**

Continued implementation of successful public and private sector strategies in support of evolving malaria drug treatment policies will be important to protect the efficacy of anti-malarial medicines and ultimately facilitate malaria elimination in Cambodia by 2025.

**Electronic supplementary material:**

The online version of this article (doi:10.1186/s12936-016-1200-2) contains supplementary material, which is available to authorized users.

## Background

Cambodia has seen a general decline in clinically diagnosed cases of malaria and case fatality over the past decade, coupled with a significant and steady decrease in overall malaria prevalence [1]. Having achieved substantial reductions in malaria morbidity and mortality since 2001, Cambodia is now making the shift from a malaria control programme to a phased elimination strategy [2, 3]. The Cambodian National Strategic Plan for elimination of malaria aims to ensure that there are no malaria deaths by 2020 and *Plasmodium falciparum* malaria is eliminated by 2020 with *Plasmodium vivax* and other forms of malaria eliminated by 2025. Malaria treatment effectiveness and elimination rely on the continued efficacy of artemisinin-based combination therapy (ACT), which has been the first-line treatment for *P. falciparum* malaria in Cambodia since 2000.

The malaria situation is complex in Cambodia due to variance in malaria burden by geographic area and mobility of at-risk populations. Moreover, Cambodia is well documented as the epicenter of multi-drug resistant malaria [4] which poses a major impediment to disease containment and eradication. Delayed parasite clearance following treatment with artemisinin and artemisinin-based combination was first detected in western Cambodia in 2007, and is now confirmed across the Greater Mekong Subregion (GMS) including foci in Laos, Myanmar, Thailand and Vietnam [3, 5–8]. As resistance to earlier anti-malarial medicines spread from this region to Africa and other parts of Asia through parasite migration, there is a serious concern that a similar scenario may occur with artemisinin resistance [5, 9–11]. The loss of ACT to resistance is highly problematic given there is no other treatment available with the same efficacy and tolerability [12], lending to the fear of a malaria resurgence that could undo a decade of progress in control as well as threaten the feasibility of elimination [13].

### Cambodia’s malaria campaigns and interventions

**Fig. 1:**
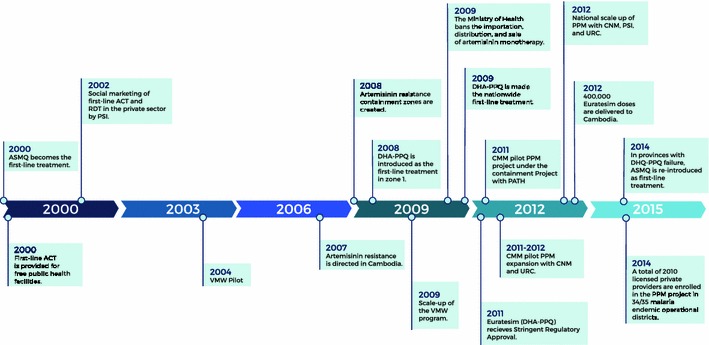
Timeline of events in Cambodia (2000–2014)

In response to the development of multi-drug resistant malaria, Cambodia designed and implemented a number of policy and strategy changes to improve coverage of appropriate case management (see Fig. 1). Dating back to 2000, Cambodia became the first country to switch its first-line national malaria treatment policy for *falciparum* malaria to an ACT of artesunate and mefloquine (ASMQ) and to stipulate diagnostic testing prior to treatment. Since 2000, the Cambodian government provided first-line ACT for free in public health facilities and parasitological diagnosis was promoted through the introduction of rapid diagnostic tests (RDT) and strengthening the capacity of skilled microscopists.

Private sector engagement in the provision of diagnosis and ACT treatment for malaria is central given that data confirms that most febrile people in Cambodia seek treatment exclusively from private facilities [14, 15]. In lieu of this, since 2002 a social marketing programme of subsidized first-line ACT and RDT was initiated through Population Services International (PSI) to improve the availability and quality of malaria diagnosis and treatment in this sector [30, 31]. The social marketing programme was designed to directly mirror the public sector, by procuring the same ACT and RDT as the national programme. As a means to ensure compliance with national treatment guidelines among private providers, sales representatives and medical detailers were also employed to provide training, counseling, and support on the appropriate use of ACT and RDT. By 2013, the PSI social marketing programme was actively reaching around 1500 outlets per month through sale representatives and around 500 pharmacy operated facilities through medical detailers.

In 2004, the Cambodia National Centre of Entomology, Parasitological and Malaria (CNM) piloted the Village Malaria Worker (VMW) programme to provide diagnosis and treatment among remote communities through a community-based health workforce, which was expanded and extended in 2009 to the highest-risk villages and remote areas. By 2014 the VMW programme was providing early diagnosis and treatment for malaria to over 1,600 villages and 130 mobile communities across 17 provinces [16].

Over the years there have been a series of changes in the National Treatment Guidelines following recommendations from the World Health Organization (WHO) to change treatments when resistance to the drug is detected. In 2008, due to high failure rates with ASMQ, the first-line treatment for uncomplicated *P. falciparum* malaria was changed from co-blistered ASMQ to fixed-dose dihydroartemisinin-piperaquine (DHA-PPQ) in specific areas of the country where artemisinin resistance had been identified; DHA-PPQ became the nationwide first-line treatment for both *P. falciparum* malaria and *P. vivax* malaria in 2010.

In 2009, the Cambodian government took significant steps to regulate the pharmaceutical industry, and banned the importation, manufacturing, registration and sale of oral artemisinin monotherapies [12]. To enforce the move, the Ministry of Health (MoH) created a new cadre of law enforcement officers, providing them with authority to crack down on illegal and substandard medicines. This included the recruitment of 200 new officials, also known as the ‘Justice Police’, as well as training of over 400 police to enable authorities to identify oral artemisinin monotherapy and enforce the ban within the Cambodian marketplace. The government also used mystery clients to further identify outlets selling any oral artemisinin monotherapy, and instituted a system of licensing drug outlets and using the Justice Police to regularly check for and confiscate fake, substandard or banned drugs. In addition, an international collaboration led by INTERPOL succeeded in shutting down a major Chinese producer of fake artemisinin that had flooded the markets throughout the GMS. These efforts were also supported with a communication campaign to educate private providers about the dangers of oral artemisinin monotherapy.

Since the original crack down of unlicensed private providers in 2009, CNM also partnered with the Ministry of Interior Anti-Economic Crime Police and the Department of Drugs and Food to intensify enforcement and regulatory activities in the private health sector [17]. Private outlets including pharmacies and drug shops were progressively registered, and it was estimated that unlicensed outlets in the country decreased from 1081 in November 2009 to only 28 in July 2011 [16].

In 2011, as a means to further engage and strengthen the private and public sector, CNM and the MoH finalized operational guidelines for implementing public private partnership (PPM) programme activities [17]. Similar to PSI’s social marketing programme, the PPM strategy provided ACT and RDT commodities, training to improve private providers’ case management skills, as well as routine supervision visits. Coordination between the public and private sectors was also advanced: a provider database of licensed outlets was created and routine malaria case-load data collection from all outlets was prioritized. A gradual expansion of the PPM programme led to national scale up in 2012, in collaboration with PSI and University Research Council (URC), and has now been extended to over 1000 providers registered across most of the malaria endemic districts. By the end of 2013, there were 10 Operational Districts (OD) under CNM, 3 under URC and 9 under PSI for the PPM programme.

There is a lack of documentation on the extent to which these changes to malaria case management policy and strategy have led to changes in the performance of Cambodia’s anti-malarial public and private sector markets. This is of particular importance given Cambodia’s recent commitment to eliminate all types of malaria by 2025 and persistent work over the past decade to contain and prevent the spread of artemisinin resistance. The primary aim of this paper is to use ACTwatch outlet survey trend data to demonstrate how changes in Cambodian policy have led to shifts in anti-malarial markets over time. Specifically the paper presents evidence from ACTwatch outlet surveys conducted in 2009, 2011, 2013 regarding the availability and market share of anti-malarials and malaria testing availability in the public and private sector.

## Methods

The ACTwatch project is a multi-country research project implemented by PSI and was launched in 2008. The goal of the ACTwatch project is to provide timely, relevant, and high quality anti-malarial market evidence to inform and monitor national and global policy, strategy, and funding decisions for improving malaria case management. Standardized tools and approaches are employed to provide comparable data across countries and over time. These approaches have been documented elsewhere in detail [15, 18–21]. One key component of the project is to collect outlet survey data to understand anti-malarial supply side issues, and to address key indicators such as: what types of outlets stock anti-malarials, what is the market share of different classes of anti-malarials and how does this differ between the public and private sector. Indicators are typically presented for both the public and private sector. Throughout the years, the project has expanded to include the same information on a full range of RDT. In short, the ACTwatch outlet survey studies are designed to provide contemporary malaria market data from all public and private sector outlets providing malaria testing or treatment to patients or consumers.

### Sample

As part of the ACTwatch project, three rounds of nationally-representative cross-sectional malaria medicine outlet surveys were conducted in Cambodia 2009 (June–July); 2011 (June–August); and 2013 (September–October). Data collection coincided with the Cambodia rainy season when malaria transmission is highest, generally beginning in late May and ending in December.

### Design and sampling

A nationally representative sample of outlets providing anti-malarials to patients was selected for each survey round. Each survey was stratified to deliver separate estimates for the zones defined by CNM to guide policies and programmes to address artemisinin resistance. Non-malaria endemic areas were excluded from the sampling frame.

The study was powered to detect a minimum of a 20-percentage point change in availability of first-line ACT among anti-malarial stocking outlets between each round. Based on the desired number of anti-malarial stocking outlets within each round and assumptions about number of anti-malarial stocking outlets per commune, a sample of communes was selected within each research domain with probability proportional to population size.

Within each selected commune all outlets with the potential to provide anti-malarials to patients were sampled (see Table 1 for a description of the outlet types). Outlets that did not serve the general public (e.g. military facilities) were excluded from the study. The boundary for the census of public health facilities was extended to the district level for over-sampling of these outlet types.

**Table 1:** Outlet types and definitions

Sector and facility type	Outlet types
Public Sector	
Public health facilities	Referral hospitals, health centers, sub-health centers, former district hospitals, and health posts
Village Malaria Worker	VMW are community-based volunteers equipped with anti-malarial treatment and malaria blood testing
Private Sector	
Private for-profit health facilities	Private for-profit health facilities including private hospitals, clinics, cabinets and diagnostic laboratories
Pharmacies	Pharmacies are licensed and regulated by a national regulatory authority, and are staffed by pharmacists and qualified health practitioners. These include clinical pharmacies, pharmacies, depot A, and depot B
Drug stores	Drug stalls in rural markets and shops that primarily sell medicines. These outlets are not guaranteed to be staffed by qualified health dispensers/practitioners and not typically licensed by a national regulatory authority
General retailers	Grocery stores and village shops
Itinerant drug vendors	Mobile providers found primarily in rural areas, typically working within a radius of their home. They are not registered with any national regulatory authority

A screening questionnaire was administered to all outlets with the potential to distribute anti-malarials to patients. Outlets were eligible for a provider interview and malaria product audit if they met at least one of the study criteria: (1) one or more anti-malarials reportedly in stock the day of the survey; (2) one or more anti-malarials reportedly in stock within the 3 months preceding the survey. A new eligibility criteria was included in the 2013 survey to screen for the provision of malaria blood testing (microscopy or RDT).

### Training and fieldwork

For all survey rounds interviewer training was provided over a minimum of 6 days, using standardized training materials. The training was implemented in collaboration with the CNM and focused on outlet identification, informed consent procedures, and the procedures for completing the questionnaire. An additional two-day training was provided for quality controllers and supervisors.

Field workers were provided with a list of the selected communes and maps that illustrated their administrative boundaries, in addition to lists of public health facilities and pharmacies obtained from relevant authorities. Snowball sampling was also used by field workers to identify facilities that were not on the official lists. In each selected cluster, fieldworkers conducted a census of all outlets that had the potential to provide anti-malarials by traveling systematically throughout the entire commune and approaching all outlets eligible for screening. For each outlet that was identified during the census, the outlet type and location were noted, along with its longitude and latitude coordinates (using hand-held global positioning units). A fieldworker then approached the outlet’s main provider or owner and invited him or her to participate in the study. Providers who agreed to participate were asked the screening questions to determine eligibility. An interview with the staff member who was most likely to sell or prescribe medications was conducted. The interview was carried out in Khmer.

During data collection, approximately 80 % of all questionnaires were reviewed by the team supervisor and 15–20 % of all outlets were revisited by a supervisor or/and quality controllers for quality control checks.

### Measures

A structured questionnaire, which included an anti-malarial audit and RDT audit, was used for the outlet survey interviews. Following informed consent procedures, an audit of all available anti-malarial medicines and RDT was first conducted. To complete the audit, providers were asked to show the interviewer all anti-malarial brands that were currently available in the outlet. Using the audit sheet, the interviewer then recorded the product information for each anti-malarial brand in the outlet, including formulation, brand name, active ingredients and strengths, manufacturer and country of manufacture. Following this, the provider was asked, for each anti-malarial in stock, the unit cost of that medicine and amount distributed to individual patients or consumers in the last week (see Additional file 1 for an example of the audit sheet). A similar pattern was repeated for outlets stocking RDT in 2011 and 2013, using the RDT audit. The RDT audit information included brand name, manufacturer, country of manufacture, reported sale/distribution in the week preceding the survey, retail price, and wholesale price (see Additional file 2 for an example of the RDT audit sheet). In addition to the anti-malarial and RDT product audit, a series of questions were administered to the senior-most provider responsible for diagnosis and/or medicine prescription. The questions assessed malaria case management knowledge and practices as well as provider training and qualifications as well as the availability of malaria microscopy.

The 2009, 2011 and 2013 outlet survey protocols received ethical approval from the National Ethics Committee for Health Research in Cambodia. Provider interviews and product audits were completed only after administration of a standard informed consent form and provider consent to participate in the study. Providers had the option to end the interview at any point during the study. Standard measures were employed to maintain provider confidentiality and anonymity.

### Data analysis

Double data entry was conducted using Microsoft Access (Microsoft Corporation, Redmond, Washington, USA) with built-in range and consistency checks. Entered data were triangulated with questionnaires, field supervision notes and daily activity logs filled by each interviewer in the field. Data were analysed across survey rounds using Stata (StataCorp College Station, TX). Stata survey settings were used to account for the stratified and clustered sampling strategy. Results were adjusted by sampling weights. Sampling weights were calculated as the inverse of the probability of commune selection.

Standard indicators were constructed according to definitions applied across the ACTwatch project and have been described elsewhere [20, 21]. Briefly, anti-malarials identified during the outlet drug audit were classified according to information on drug formulation, contents and strengths with supporting information including brand or generic name and manufacturer. Among outlets stocking anti-malarials (i.e. those outlets that had at least one anti-malarial in stock on the day of survey), variables were created to indicate availability by type, including the broad category of ACT, as well as specific categories including all ASMQ, DHA-PPQ, oral artemisinin monotherapy, non-oral artemisinin monotherapy, and non-artemisinin monotherapy (such as chloroquine).

Availability of anti-malarials was measured as the proportion of outlets with at least one anti-malarial in stock, among all censused outlets. Availability was determined according to the anti-malarial packages that were found in the outlet and then audited. The availability of specific anti-malarial categories, restricted to those outlets that had anti-malarials in stock, was also calculated. Thus, for example, the availability of ACT was measured as the proportion of outlets stocking ACT, among all outlets with at least one anti-malarial in stock.

The volume of the anti-malarials recorded in the drug audit were standardized using the adult equivalent treatment dose (AETD) to allow meaningful comparisons between anti-malarials with different treatment courses. The AETD is defined as the amount of active ingredient required to treat an adult weighing 60 kg according to WHO treatment guidelines [22]. Provider reports on the amount of the drug sold or distributed during the week preceding the survey were used to calculate volumes according to type of anti-malarial: ASMQ, DHA-PPQ, other ACT, oral artemisinin monotherapy, non-oral artemisinin monotherapy and non-artemisinin monotherapy. The volume of each drug is therefore the number of AETDs that were reportedly sold/distributed during the week preceding the survey. Measures of volume include all dosage forms to provide a complete assessment of anti-malarial market shares to the consumer or patient. Audited anti-malarials with missing data required to calculate AETD (strength, amount distributed) were excluded from volumes estimation. This measure of anti-malarial volumes was then used to describe the ACTwatch indicator on market share, and is subsequently referred to as this.

## Results

A total of 7833 outlets in 2009, 18,584 in 2011 and 16,153 in 2013 were approached to participate in the surveys. Of these, over 95 % of outlets were screened for stocking anti-malarials across the three survey rounds. Among screened outlets, 11.5 % of outlets in 2009, 8.5 % of outlets in 2011 and 9.2 % of outlets in 2013 met the screening criteria and were administered the questionnaire (Table 2). The total number of anti-malarials audited were as follows: 2009, N = 2051; 2011, N = 2598 and 2013, N = 2811. An increase is observed in the number of RDT audited over time (2009, N = 653; 2011, N = 1005; 2013, N = 1267).

**Table 2:** Outlet sample composition 2009, 2011 and 2013

	2009	2011	2013
Among outlets approached	N = 7833	N = 18,584	N = 16,153
Proportion that were screened	95.9 %	96.6 %	97.5 %
Among all outlets screened	N = 7513	N = 17,923	N = 15,755
Proportion that met the screening criteria (%)	11.5	8.5	9.2
Proportion that were interviewed (%)	11.5	8.4	9.1
Among outlets screened	N	N	N
Number that meet the screening criteria			
Anti-malarial(s) in stock	865	1283	1221
Anti-malarial(s) out of stock but reportedly in stock in the previous 3 months	7	246	123
Anti-malarial(s) not in stock but blood testing available	n/a	n/a	112
Total	872	1529	1456
Number that were interviewed			
Anti-malarial(s) in stock	863	1270	1215
Anti-malarial(s) out of stock but reportedly in stock within 3 months	7	246	123
Anti-malarial(s) not in stock but blood testing available	n/a	n/a	111
Total	870	1516	1449

*n/a* screening questions regarding the availability of blood testing were not included in the 2009 and 2011 surveys

### Anti-malarial availability

**Fig. 2:**
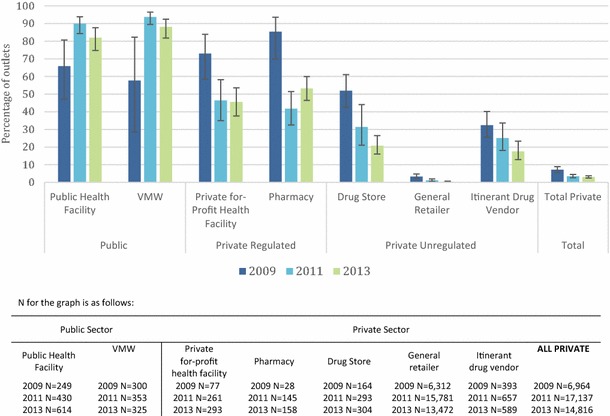
Percentage of all outlets with at least one anti-malarial in stock on the day of survey

Figure 2 shows trends in the availability of anti-malarials as the proportion of all surveyed outlets. The percentage of public health facilities with at least one anti-malarial in stock on the day of the survey increased between 2009 (65.8 %) and 2011 (90.0 %) and remained high in 2013 (82.0 %). Similar trends were found for VMW (2009 = 57.7 %; 2011 = 93.7 %; and 2013 = 88.1 %). Overall, private sector availability of anti-malarials declined over time and varied by outlet type, with private for-profit health facilities and pharmacies more likely to stock anti-malarials than other private sector outlet types. In 2013, just over half of the pharmacies (53.2 %) and 45.5 % of private for-profit facilities had anti-malarials in stock on the day of survey. Stocking rates by drug stores, general retailers and itinerant drug vendors were less than 20 %.

**Fig. 3:**
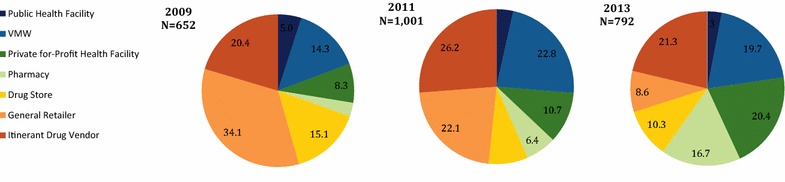
Trends in the market distribution among anti-malarial stocking outlets (2009 N = 652 outlets; 2011 N = 1001 outlets; 2013 N = 792 outlets)

Figure 3 shows the relative distribution of all outlets that had at least one anti-malarial in stock, over time. Results show a change in the diversity of outlet types stocking anti-malarials. Private sector market composition shifted over time towards increasing distribution through the private for-profit health facility and pharmacy outlet types and declining contribution from drug stores, general retailers, and itinerant drug vendors. Private for-profit facilities increased from 8 % of all outlets stocking anti-malarials in 2009 to 20 % in 2013. Similarly, pharmacies increased from 3 % of all outlets stocking anti-malarials in 2009 to 17 % in 2013. In contrast, declines were observed for general retailers, which constituted 34 % of all outlets stocking anti-malarials in 2009 and only 9 % of outlets in 2013. Similar declines were observed for drugs stores, which comprised of 15 % of all anti-malarial stocking outlets in 2009 and only 10 % in 2013.

**Fig. 4:**
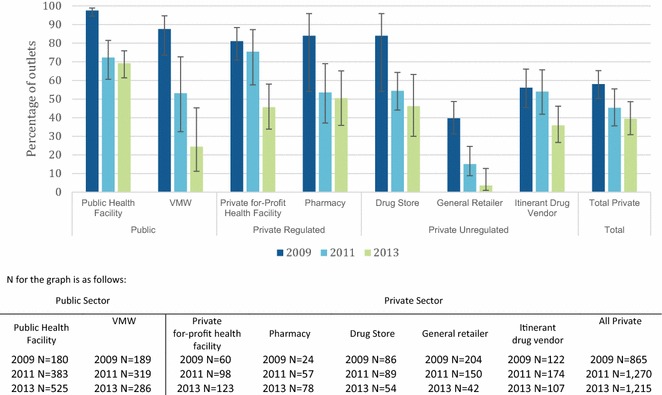
Trends in the availability of anti-malarial stocking outlets with ASMQ in stock on the day of the survey

Figure 4 show trends in the availability of different classes of the two first-line ACT (ASMQ and DHA-PPQ) as the proportion of outlets with at least one anti-malarial in stock. In 2009 when ASMQ was the first-line treatment for Cambodia, with the exception of resistance containment areas of the country (for which DHA-PPQ had been the first-line since 2008), availability was very high in anti-malarial stocking public health facilities (97.5 %) and VMW (87.6 %), as well as private for-profit facilities (87.1 %), pharmacies and drug stores (84.0 %). ASMQ was less available among general retailers (40 %) and itinerant drug vendors (~60 %) in 2009.

**Fig. 5:**
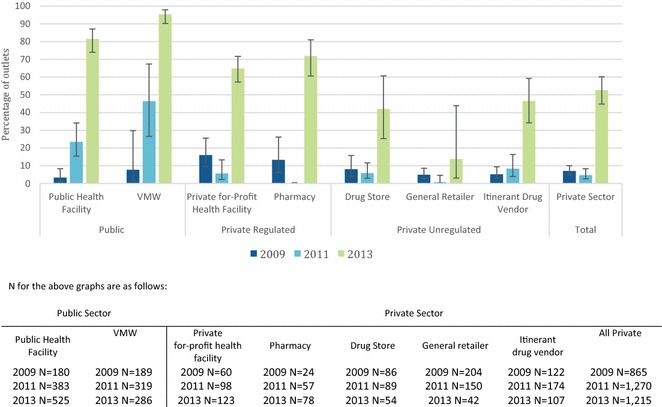
Trends in the availability of anti-malarial stocking outlets with DHA-PPQ in stock on the day of the survey

After the change in the national first-line treatment from ASMQ to DHA-PPQ in 2010, ASMQ availability declined across all outlet types, however availability of DHA-PPQ was modest among anti-malarial stocking public health facilities (23.5 %), VMW (46.4 %) and was less than 10 % across all private sector outlets in 2011 (Fig. 5). By 2013, significant increases were observed in DHA-PPQ availability across all anti-malarial stocking outlet types. The majority of anti-malarial stocking public health facilities had DHA-PPQ in stock (81.5 %), as did VMW (95.4 %), with private health facilities and pharmacies also showing high DHA-PPQ availability (64.8 and 71.9 % respectively). DHA-PPQ availability was lower among anti-malarial stocking drug stores (42.0 %), general retailers (13.7 %), and itinerant drug vendors (46.5 %).

**Fig. 6:**
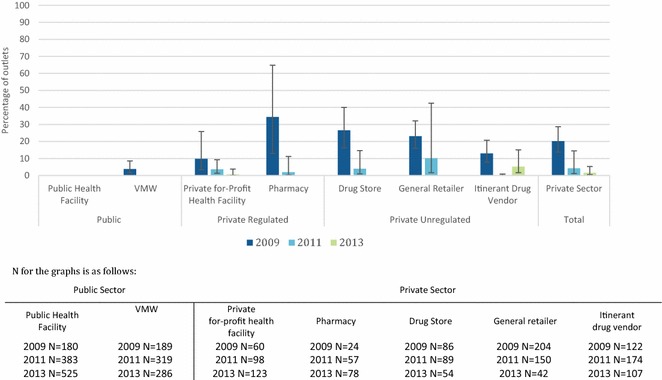
Trends in the availability of anti-malarial stocking outlets with oral artemisinin monotherapy in stock on the day of the survey

The percentage of private sector anti-malarial stocking outlets with oral artemisinin monotherapy in stock on the day of the survey decreased over time (2009 = 20.2 %; 2011 = 4.2 %; and 2013 = 1.6 % (Fig. 6). In 2013, availability had further dropped to 0.6 % among private for-profit health facilities, and 5.2 % among itinerant drug vendors. No oral artemisinin monotherapy was found in public health facilities or among VMW in 2011 and 2013.

### Anti-malarial market share

**Fig. 7:**
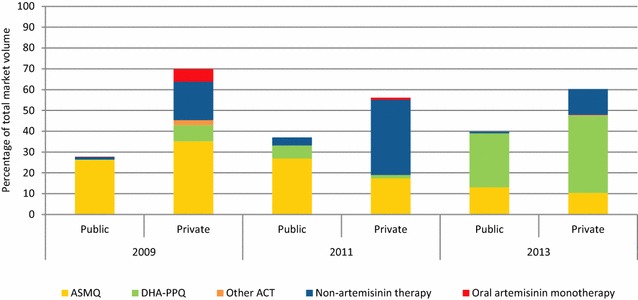
Trends in anti-malarial market share (A total of 1828.2, 919.9 and 284.0 AETDs were reportedly sold or distributed in the previous 7 days in 2009, 2011 and 2013 respectively)

Figure 7 shows the market share of different categories of anti-malarials sold or distributed in the 7 days prior to the survey over time. Public sector anti-malarial market share increased over time from 30 % in 2009 to 40 % in 2013. In the private sector, anti-malarial market share declined over time from 70 % in 2009, to 60 % in 2013. ACT accounted for 72 % of the total market share in 2009; 52 % in 2011 and 87 % in 2013.

In terms of the market share of the first-line treatment in 2009 ASMQ was the most commonly distributed anti-malarial, with the public sector distributing little else. In 2011, ASMQ continued to be the most commonly distributed anti-malarial, with the new first-line DHA-PPQ accounting for less than 10 % of the total market share, and the majority of this distributed in the public sector. Increases in the market share of chloroquine in the private sector were also notable, comprising 21.7 % of the total market share in 2011. During the 2013 survey, most of the ACT either sold or distributed nationally was DHA-PPQ (62.8 %). Market share of ASMQ was lower than previous rounds (23.4 %). Chloroquine made up the largest market share of non-artemisinin therapies, accounting for 12.4 % of the market share in 2013, and found exclusively in the private sector.

**Fig. 8:**
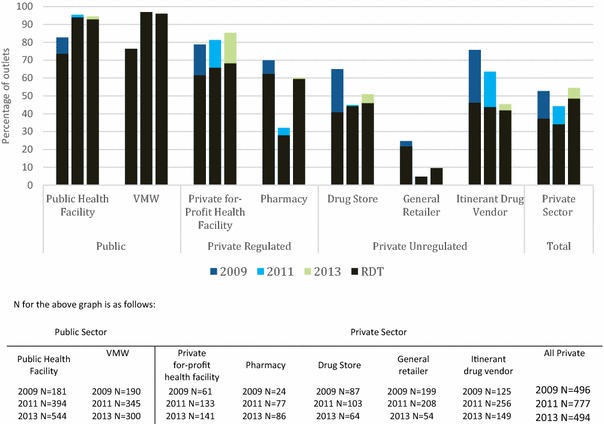
Percentage of anti-malarial stocking outlets with malaria blood testing available

Oral artemisinin monotherapy accounted for 6 % of total anti-malarial market share in 2009 and 1 % in 2011, but was no longer reportedly sold/distributed in 2013.

### Malaria diagnostics

The percentage of anti-malarial stocking outlets with malaria blood testing available (RDT or microscopy) remained high over time among public health facilities (over 80 % in 2009, and over 95 % in 2011 and 2013). Among VMW this also increased between 2009 from 73.6 % to over 92 % in 2011 and 2013. In 2013, over 90 % of anti-malarial stocking public health facilities and VMW had malaria blood testing available (Fig. 8).

Overall trends in blood testing availability among other outlet types suggest lower availability in the private sector (2009 = 52.7 %; 2011 = 44.2 %; and 2013 = 54.3 %). However, in 2013, 85.3 % of anti-malarial stocking private for-profit facilities, 60.1 % of pharmacies and 50.9 % of drugs stores had malaria blood testing available. Only 10 % of anti-malarial stocking general retailers had malaria testing available.

Trends in the percentage of anti-malarial stocking outlets with malaria microscopy show a slight decline in availability over time, with lower levels of availability observed in 2013 (<35 %) in both public and private sector outlets (Additional file 3). Availability of malaria RDT increased among anti-malarial stocking public health facilities and VMW between 2009 and 2011 and remained high in 2013 (93 and 96 % respectively). RDT availability remained moderate (~50 %) among anti-malarial stocking private outlets with the exception of general retailers.

## Discussion

Shifts in malaria case management policy and strategy to address anti-malarial drug resistance have been successfully implemented within the public and private sectors in Cambodia. Data from three nationally-representative outlet surveys confirm substantial improvements between 2009–2013 regarding removal of oral artemisinin monotherapy, ensuring high availability and market share of first-line ACT treatments, and a reduction in the role of drug shops and general retailers in the distribution of anti-malarials relative to other private sector outlet types such as health facilities and pharmacies. The data also illustrate wide spread availability of malaria diagnostics for confirmatory testing prior to anti-malarial treatment in the public sector, and moderate availability in the private sector. The following sub-sections discuss these changes in the context of national strategies and policies to improve malaria case management practices and artemisinin resistance containment efforts over the past 5 years in Cambodia.

### Oral AMT removal

Significant reductions and removal of oral artemisinin monotherapy across the private sector reflect effective policy changes and successful enforcement and implementation by Cambodian officials, including the MoH, Ministry of Interior, and the Regulatory Agency for Drugs and Foods [23]. Historically, artemisinins have been available as monotherapies in western Cambodia for more than 30 years, in a variety of forms and doses. Oral artemisinin monotherapies were notably less expensive than ACT in Cambodia, fueling their market availability and consumption [24]. It was also estimated that in 2007, 78 % of artemisinin use in western Cambodia consisted of monotherapy provided through the private sector [25]. The 2013 outlet survey shows that no oral artemisinin monotherapy was being sold or distributed in the week prior to the survey. Measures by the Cambodian government to stop the importation and distribution of oral artemisinin monotherapy and supportive and educative activities with private providers of malaria treatment were successful.

### Availability and distribution of first-line ACT

High availability and market share for the first-line ACT in 2013 reflect effective strategies to implement changes in national treatment guidelines. However, 2011 availability and market share of the first-line treatment is lower, particularly in the private sector, and is indicative of considerable challenges regarding the shift from ASMQ to DHA-PPQ as the first-line treatment in December 2010. These challenges are and discussed in this sub-section.

In 2010, DHA-PPQ became the first-line treatment in Cambodia. In 2011, the outlet survey data shows that in the public sector, 8 months after the changes to the treatment guidelines, around half of all VMW and one in four public health facilities had DHA-PPQ in stock, indicating relatively rapid changes, but still only moderate availability. In the private sector, availability and market share of DHA-PPQ was substantially lower as compared to the public sector. Market share data from 2011 also shows an increase in chloroquine distribution as compared to data from 2009, and despite the fact that chloroquine was removed from the national treatment guidelines in 2000. It is only in 2013 that DHA-PPQ becomes the most commonly distributed anti-malarial in both the public and private sectors.

The reasons for low availability and market share of the first-line treatment in 2011 centre around procurement regulations pertaining to purchase of medicines using international donor funds. When the first-line treatment changed in 2010, two manufacturers were producing DHA-PPQ. Neither of these manufacturers had good manufacturing practice (GMP) or stringent regulatory authority (SRA) approvals, both of which are required in order to procure anti-malarial medicines under international donor regulations. In October 2011, one brand of DHA-PPQ, Euratesim^®^, received SRA approval through the European Medicines Agency (EMA) [26] allowing a much delayed order to be placed. The 2011 outlet survey captures this contextual background, illustrating a low availability of DHA-PPQ in the public sector, and no availability in the private sector. The moderate availability in the public sector is explained by efforts by the WHO in 2010, who procured DHA-PPQ directly for CNM from the second producer. However, the private sector was faced with an unprecedented 10 month ACT stock-out [27]. It was only in July 2012, that 400,000 doses were delivered to Cambodia’s private sector [26]. Significant shifts in first-line ACT availability in both the public and private sectors are therefore only observed in the 2013 outlet survey, where DHA-PPQ was the most commonly distributed anti-malarial nationwide, and chloroquine availability and market share had dropped in the private sector.

The procurement problems faced in 2010 had detrimental effects on malaria control efforts and strategies in Cambodia that were beyond the control of the government and other national stakeholders. While the WHO was able to procure much needed first-line ACT for the public sector, the effects were devastating for the private sector where over 65 % of patients seek care when they suspect they have malaria [14, 15]. This situation demonstrates the fragility of the anti-malarial market, and illustrates how constant supply of quality-assured ACT, supported by donors, is challenging given the fact that there are only few manufacturers with the required regulatory approvals. The need to rapidly respond to evolving drug resistance further compounds the situation, and highlights the challenges that in-country stakeholders and the government face in executing fast responses on the ground.

In its continuing effort to keep one step ahead of drug resistance, Cambodia’s national treatment guidelines changed again in 2014 and currently stipulate the use of both co-formulated ASMQ and DHA-PPQ in different parts of the country as first-line drug for treatment of uncomplicated malaria. In Cambodia’s situation where bespoke, rather than off-the-shelf anti-malarials, the MoH and donors may need to consider parallel stocking systems whereby two or more anti-malarials are stocked in-country to guarantee anti-malarials are immediately on-hand depending on the drug resistance, while accepting that drug wastage will be an inevitable reality in the drive towards elimination.

### Roles and regulation for the private sector

While anti-malarial availability is declining in the private sector, private sector outlets are still responsible for substantial anti-malarial market share indicating this is an important source of treatment for febrile patients. The most recent Cambodia malaria survey (2013) also shows that over half the population exclusively obtain treatment from the private sector, and up to 40 % first seek treatment from outlets such as drug shops, retailer outlets and itinerant vendors [14]. The 2009, 2011 and 2013 outlet surveys show that the performance of the private sector over time has improved, with most of these outlet types distributing the first-line treatment in 2013. It is also notable that the private sector market composition has shifted over time towards an increasing contribution from private health facilities and pharmacies, and a declining contribution from drug stores and general retailer outlets—outlets which typically operate without formal licensing or registration, and are not formally regulated.

Active and supportive regulation has also had a key role to play in improving the performance of the market for malaria treatment. This has included active engagement by CNM with the private sector through PSI to ensure the availability of high quality first-line ACT treatments and RDT in the private sector through social marketing techniques. Sales data indicate that PSI is the largest importer and distributor of ACT in the country and quite likely one of the largest pharmaceutical distributors overall [28]. PSI sales data indicate that in 2009, around 280,000 ACT doses were distributed and 107,600 in 2013. Sales were much lower in 2011 given the aforementioned fist-line DHA-PPQ stock-out, with only 64,000 ACT doses distributed, and thus explaining some of the drop in ACT availability and market share in 2011. In 2009, PSI was distributing ACT and RDT to over 1700 outlets such as clinics and hospitals, pharmacies, and drug stores, which can also serve as wholesalers from which other outlets can procure medicines [29]. Tightened regulation also meant that only registered outlets were allowed to receive ACT and RDT through PSI, perhaps helping to explain the overall drop in anti-malarial availability across different private outlet types, though this could also reflect decreasing malaria caseload, lending to a disincentive among private providers to stock anti-malarial medicines.

The changing performance of the private sector is also reflected in the increasing number of licensed private providers and intensified enforcement and regulatory activities in the private health sector [17]. Private outlets have been progressively registered since 2011, with over 2000 private outlets across the country currently registered by Department of Drugs and Food.

Efforts to engage the private sector also show an increase in the number of licensed private providers under the PPM programme network as a means to improve the quality of malaria case management and to ensure malaria case load data is reported up to CNM. It is promising that under Cambodia’s 2016–2020 malaria elimination plan, the PPM programme will be scaled up across the 45 malaria endemic ODs to reach all licensed providers. The government has plans to map both licensed and unlicensed providers. Unlicensed providers will be encouraged to obtain the required license enabling them to be eligible for joining the PPM programme, and CNM also plans to strengthen monitoring and supervision of the programme and will supply ACT and RDT directly to private providers in their implementation areas. Additionally, there are plans to restrict the import or sale of any anti-malarial not recommended as per national policy and thus further increasing the availability and quality of case management services in the private sector [2]. Mystery clients may be a particularly useful survey method to help to flush out monotherapies and other illegal medicines that would have otherwise be hidden by providers, given the increase in regulation of the private sector outlets. In addition to tightened regulation of the private sector market, efforts must continue to engage the general population regarding the appropriate sources of treatment for febrile illness.

### Confirmatory testing prior to anti-malarial treatment

Adherence to case management policy stipulating confirmatory testing prior to treatment is currently facilitated by high availability of malaria blood testing among public and private-for-profit health facilities and VMW, and moderate availability among pharmacies and drug shops. The data also point to a marked increase in RDT availability across the different outlet types, including public health facilities. In fact, public health facility caseload data suggested a marked shift from malaria microscopy to RDT. Between 2008 and 2010 the number of slides taken annually in health facilities decreased from 88,742 to 82,565. At the same time the number of RDT used in public health facilities increased from 46,989 to 178,364, indicating a shift to reliance on RDT for the majority of confirmed diagnoses [27]. This increase may also be facilitated by the introduction in 2009 of a Combo RDT in the public and private sector, which detects both *falciparum* as well as *vivax* malaria. VMW, which only stock RDT also show significant improvements in RDT availability after the expansion in 2009. The importance of VMW programme is also reflected in findings from a population based survey which suggests that the likelihood of a patient receiving an RDT in areas where the VMW programme is present increases 11-fold as compared to those areas where the VMW programme is not present [25]. Furthermore, it is estimated that VMW diagnosed and treated more than half of malaria cases recorded in the public sector in 2014 and 100 % of these cases are confirmed by RDT, suggesting the importance of maintaining constant supply to these providers.

Observing the private sector, availability of malaria is somewhat variable, but overall stocking rates are much higher than observed in other ACTwatch countries [30]. These findings reflect the recognition and the importance of the private sector by the Cambodian government, and strategies to manage diagnosis, treatment, and refer within this sector. Since 2000, the government policy has been to actively promote the use of tests prior to treatment for malaria. Since 2002, the government policy has allowed malaria diagnosis using RDT to be introduced into selected private health facilities through the social marketing approaches implemented by PSI [28, 29]. PSI recommends a very low RDT price to encourage providers to test every febrile patient, and to incentivise patients to get treated. As such, for over a decade the Cambodian private sector has been authorized to test, and treat, cases of uncomplicated malaria. In 2009 over 350,000 RDT were distributed by PSI in the country, and over 400,000 in 2010 and 2012, and 475,670 in 2013. This collaboration between CNM and PSI has helped to ensure the readiness and adherence of the private sector to adhere to national guidelines.

Given that one of the cornerstones of malaria elimination is parasitological confirmation of all suspected malaria cases by either microscopy or RDT [31], outlet survey results point to the fact that current rates of diagnostic availability are sub-optimal among private sector anti-malarial stocking outlets, though it is acknowledge that RDT availability may be higher among those outlets that are part of the PPM programme. Results also suggest that anti-malarials continue to be distributed by outlets where confirmatory testing is not available in the private sector. In support of this, evidence from the Cambodia malaria survey in 2013 revealed that only 11 % of febrile patients received a confirmatory diagnostic test, suggesting there is widespread need to not only promote the availability of tests, but also ensure they are administered [32]. These findings point to two issues that relate to the success of Cambodia’s immediate elimination strategy in the context of diagnosis. First is the assurance of wide spread availability of diagnostic testing to ensure 100 % parasitological diagnosis of all suspected malaria cases by 2016. This may be achieved by tapping into on-going PPM programme efforts and continued efforts to socially market RDT. Second is the need to ensure that all patients seeking treatment for fever are tested prior to being administered medication. Given the current disconnect, there is a need to understand the demand drivers for adoption of diagnosis among health care providers in the private sector.

## Limitations

Despite the study’s strengths, the ACTwatch outlet survey data have several limitations, which have been documented elsewhere in detail [19, 21, 32]. Specific to the surveys in Cambodia: (i) providers may have had incentives to mis-report certain information. The ban on the sale of oral artemisinin monotherapy and tightened regulation of the private sector may have made providers wary to report availability of certain types of anti-malarials, or in the case of outlets that had not been licensed, to report any availability of anti-malarial medicines. Although interviewers stressed that they were not connected with any regulatory body, it is acknowledged that anti-malarial stocks may have been under-reported as found by others using similar census methods [33]. (ii) Data were only collected at three time points over 5 years and estimates of availability apply to the survey period. These estimates do not reflect the extent to which anti-malarial medicine availability fluctuated due to stock-outs that may have occured more than 3 months prior to the data collection. In addition, given that data were not collected during the same time period across the survey rounds, seasonal variation regarding availability of anti-malarials and volumes may be expected. In particular, given that the 2013 data were collected towards the end of the malaria season from September to October, anti-malarial availability and sales volumes may have been lower than as compared to the other survey rounds where data collection started in June. (iii) While a methodological strength of the survey is the full census of outlets in selected clusters, there were a number of practical implementation challenges with this method. Heavy rains, flooding and difficult terrain made it challenging for field workers to conduct a full census in the clusters to screen every outlet. (iv) While the study addressed key anti-malarial market indicators, assessment of drug quality or storage conditions of the anti-malarials was not determined. Providing additional information on the quality of anti-malarials, as well as the identification of any falsified anti-malarials, would have provided a more comprehensive view of the anti-malarial landscape. That said, recent drug quality evidence collected from Cambodia’s private sector found no falsified medicines, though around one in three medicines were deemed poor quality, and around one tenth were past their expiry date [33]. (v) Finally, the outlet survey methodology is most useful for measuring malaria diagnostic testing and medicine availability and relative distribution of anti-malarial treatments. However the methodology is less useful for measuring appropriate malaria case management for individual cases. This includes confirmatory testing for each suspected case, prescription based on test results, and adherence to full course treatments. Tracking appropriate case management for all suspected malaria cases will be important for informing and monitoring progress towards malaria elimination in Cambodia.

## Conclusion

Multiple drug policy changes and effective enforcement strategies have been required in Cambodia to respond to artemisinin drug resistance and to drive progress towards *P. falciparum* elimination. These drug policy changes have been implemented by public and private sector partners with success, including the removal of oral artemisinin monotherapy and changes in first-line therapy. The vast majority of anti-malarials distributed in Cambodia through public and private outlets are now first-line ACT. Adherence to case management policy stipulating confirmatory testing prior to treatment is currently facilitated by high availability of malaria blood testing among public and private health facilities and VMW and moderate availability among pharmacies and drug shops. Continued implementation of successful public and private sector strategies in support of evolving drug policies will be important to protect the efficacy of anti-malarial medicines and ultimately facilitate *P. falciparum* elimination in Cambodia. Ensuring full access to quality diagnosis and treatment services among private sector providers will be essential to the country’s elimination efforts. Overcoming procurement bottlenecks will allow the country to respond rapidly to emerging drug resistance by facilitating the immediate availability of malaria first-line treatment. Future ACTwatch outlet surveys in Cambodia, as well as Laos, Thailand, Myanmar and Vietnam will provide standardized measures of anti-malarial market composition, readiness and performance for appropriate case management. This evidence will contribute towards building an enabling environment at national, regional and global levels for progress towards malaria elimination.

## Additional files

10.1186/s12936-016-1200-2 ACTwatch Drug Audit for Tablet, Suppository and Granuales, Cambodia 2013.
10.1186/s12936-016-1200-2 RDT audit, Cambodia 2013.
10.1186/s12936-016-1200-2 Availability of malaria blood testing among anti-malarial stocking outlets*, by outlet type, across survey rounds.
