# Supplementary figures and images for: Evidence of successful malaria case management policy implementation in Cambodia: results from national ACTwatch outlet surveys
Source: Malar J. 2016 Apr 8;15:194. doi: 10.1186/s12936-016-1200-2 (PMC4826540; doi:10.1186/s12936-016-1200-2)

Supplementary file 1: ACTwatch Drug Audit for Tablet, Suppository and Granuales, Cambodia 2013


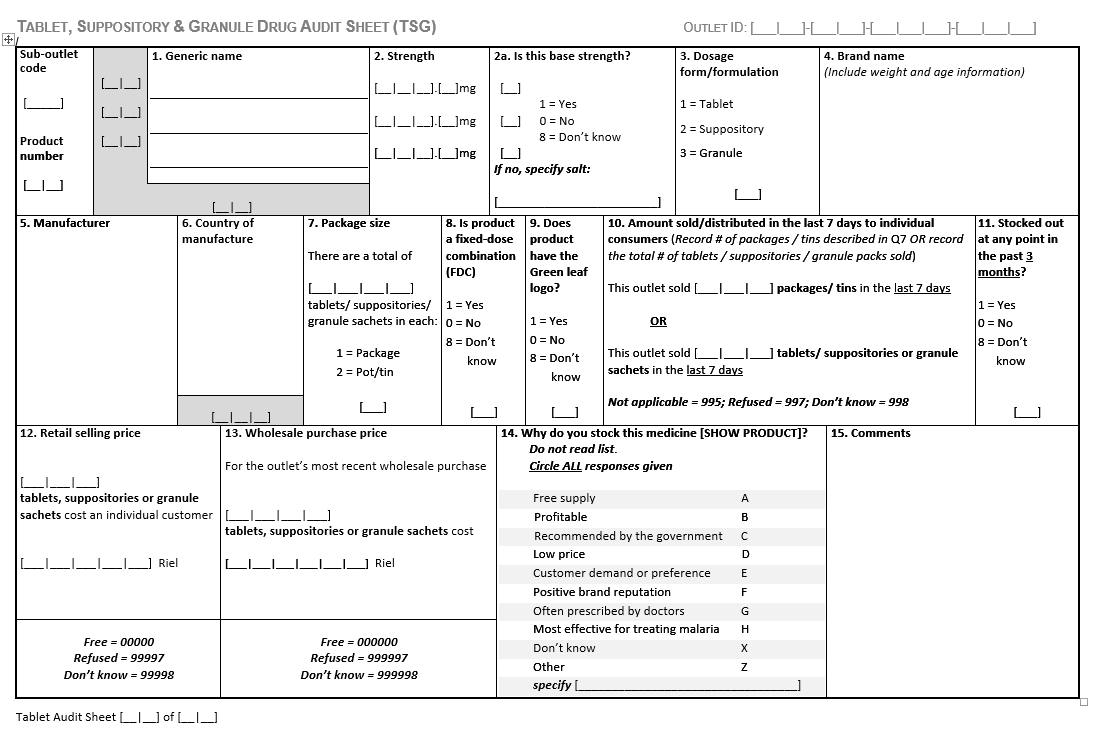

Supplement: Supplementary file 1 — 10.1186/s12936-016-1200-2 ACTwatch Drug Audit for Tablet, Suppository and Granuales, Cambodia 2013. [file 12936_2016_1200_MOESM1_ESM.docx]
