# Supplementary material for: Evidence of successful malaria case management policy implementation in Cambodia: results from national ACTwatch outlet surveys
Source: Malar J. 2016 Apr 8;15:194. doi: 10.1186/s12936-016-1200-2 (PMC4826540; doi:10.1186/s12936-016-1200-2)
Supplement: Supplementary file 2 — 10.1186/s12936-016-1200-2 RDT audit, Cambodia 2013. [file 12936_2016_1200_MOESM2_ESM.pdf]

Supplementary file 2, RDT Audit, Cambodia 2013

Clinic code<br>[_____]<br>_______<br>Product number<br>[__|__]	**1. Brand name**<br>**(Include the terms such as Pf, Pv, Pan if visible on the pack)**	**2. Manufacturer**	**3. Country of Manufacture**<br><br>[___|___|___]	**4. Lot Number**	**5. Number of tests sold/ distributed /used in the last 7 days to individual consumers**<br>*(Record total # of tests)*<br>This outlet sold or distributed<br><br>[___|___|___|___] tests in the last 7 days<br><br>***Refused = 9997 ; Don't know = 9998***

**6a. Has this test been stocked out at any point in the past 2 weeks?**<br>1 = Yes<br>0 = No<br>8 = Don't know<br><br>[___]	**6b. Has this test been stocked out at any point in the past 3 months?**<br>1 = Yes<br>0 = No<br>8 = Don't know<br><br>[___]	**6c. Has this test been stocked out for at least one week at any point in the past 3 months?**<br>1 = Yes<br>0 = No<br>8 = Don't know<br><br>[___]	**7. Price for adults**<br>**What is the total cost of an RDT for an adult:** [___|___|___|___|___] Riel<br>**Of this total, what is the cost for the test kit only:** [___|___|___|___|___] Riel<br>**Of this total, what is the cost for consultation or other fees:** [___|___|___|___|___] Riel	**8. Wholesale purchase price**<br>For the outlet's most recent wholesale purchase:<br><br>[___|___|___|___] tests cost<br><br>[___|___|___|___|___] Riel	**9. Comments**
			Free = 00000; NA = 99995; Refused = 99997; Don't Know		

RDT Audit Sheet [___|___] of [___|___]
