# Supplementary material for: Evidence of successful malaria case management policy implementation in Cambodia: results from national ACTwatch outlet surveys
Source: Malar J. 2016 Apr 8;15:194. doi: 10.1186/s12936-016-1200-2 (PMC4826540; doi:10.1186/s12936-016-1200-2)
Supplement: Supplementary file 3 — 10.1186/s12936-016-1200-2 Availability of malaria blood testing among anti-malarial stocking outlets*, by outlet type, across survey rounds. [file 12936_2016_1200_MOESM3_ESM.docx]

Supplementary file 3: Availability of malaria blood testing among anti-malarial stocking outlets*, by outlet type, across survey rounds

| Availability of malaria blood testing among anti-malarial stocking outlets*, by outlet type, across survey rounds | | | | | | | | | | |
| --- | --- | --- | --- | --- | --- | --- | --- | --- | --- | --- |
|  | Public  Health  Facility | Community Health Worker | **ALL**  **Public / Not for-profit** | Private  for-profit  HF | Pharmacy | Drug Store | General retailer | Itinerant  drug vendor | **ALL**  **Private** | **ALL**  **Outlets** |
|  | (91.3, 96.6) | (92.6, 98.0) | (93.3, 97.0) | (78.5, 90.2) | (45.7, 73.0) | (34.9, 66.8) | (2.0, 35.8) | (32.6, 58.8) | (44.4, 64.4) | (57.5, 72.4) |
| Microscopic blood tests | 2009 N=181  2011 N=391  2013 N=541 | 2009 N=189  2011 N=341  2013 N=300 | 2009 N=370  2011 N=732  2013 N=841 | 2009 N=61  2011 N=133  2013 N=141 | 2009 N=24  2011 N=77  2013 N=86 | 2009 N=87  2011 N=103  2013 N=64 | 2009 N=199  2011 N=208  2013 N=54 | 2009 N=125  2011 N=256  2013 N=149 | 2009 N=496  2011 N=777  2013 N=494 | 2009 N=866  2011 N=1,509  2013 N=1,335 |
| 2009 | 38.1 | 2.1 | 17.0 | 40.2 | 23.9 | 32.3 | 4.1 | 43.8 | 24.1 | 21.9 |
|  | (27.1, 50.4) | (0.5, 9.0) | (13.0, 22.0) | (29.4, 52.0) | (12.1, 41.7) | (19.5, 48.3) | (2.3, 7.0) | (33.1, 55.1) | (18.9, 30.2) | (18.0, 26.5) |
| 2011 | 22.5 | 0.5 | 9.1 | 31.4 | 8.4 | 9.3 | 0.9 | 23.5 | 15.3 | 13.5 |
|  | (18.8, 26.6) | (0.2, 1.6) | (6.6, 12.3) | (19.1, 47.1) | (3.3, 19.6) | (4.4, 18.7) | (0.2, 5.2) | (13.2, 38.4) | (9.7, 23.4) | (9.1, 19.7) |
| 2013 | 19.5 | 11.0 | 14.3 | 35.3 | 11.6 | 14.3 | 0.0 | 11.7 | 16.5 | 15.9 |
|  | (15.3, 24.6) | (4.6, 24.0) | (9.2, 21.7) | (27.7, 43.8) | (5.0, 24.7) | (6.0, 30.2) | - | (6.4, 20.4) | (12.4, 21.5) | (12.5, 20.0) |
| Rapid diagnostic tests (RDT) | 2009 N=180  2011 N=394  2013 N=544 | 2009 N=190  2011 N=345  2013 N=300 | 2009 N=370  2011 N=739  2013 N=844 | 2009 N=61  2011 N=133  2013 N=141 | 2009 N=24  2011 N=77  2013 N=86 | 2009 N=87  2011 N=103  2013 N=64 | 2009 N=199  2011 N=207  2013 N=54 | 2009 N=125  2011 N=256  2013 N=149 | 2009 N=496  2011 N=776  2013 N=494 | 2009 N=866  2011 N=15,15  2013 N=1,338 |
| 2009 | 73.6 | 76.3 | 75.2 | 61.5 | 62.3 | 40.8 | 21.7 | 46.2 | 37.2 | 48.9 |
|  | (59.4, 84.1) | (62.2, 86.3) | (66.0, 82.5) | (46.8, 74.3) | (30.5, 86.1) | (26.5, 56.8) | (11.0, 38.5) | (35.8, 56.9) | (28.2, 47.3) | (40.0, 57.9) |
| 2011 | 93.9 | 96.9 | 95.7 | 65.7 | 27.9 | 44.2 | 4.8 | 43.7 | 34.0 | 51.7 |
|  | (90.1, 96.3) | (93.4, 98.6) | (93.2, 97.3) | (54.3, 75.5) | (15.5, 45.0) | (29.5, 59.9) | (2.7, 8.3) | (31.7, 56.5) | (28.5, 40.0) | (45.0, 58.4) |
| 2013 | 92.8 | 96.0 | 94.7 | 68.2 | 59.4 | 45.9 | 9.6 | 41.8 | 48.4 | 60.6 |
